# Exploiting Arch-like Foot Structure for Knee-Extended Walking in Bipedal Robots

**DOI:** 10.3390/biomimetics10020096

**Published:** 2025-02-09

**Authors:** Yudi Zhu, Zhiyuan Liang, Jun Tang, Yunfeng Hou, Qingdu Li, Jianwei Zhang

**Affiliations:** 1 School of Optoelectronic Information and Computer Engineering, University of Shanghai for Science and Technology, Shanghai 200093, China; 221240082@st.usst.edu.cn (Y.Z.); zyuanliang@126.com (Z.L.); 211240187@st.usst.edu.cn (J.T.); yunfenghou@usst.edu.cn (Y.H.); 2 Institute of Machine Intelligence, University of Shanghai for Science and Technology, Shanghai 200093, China; 3 Zhongyu Embodied AI Laboratory, Zhengzhou 450000, China; 4 Department of Informatics, University of Hamburg, 20146 Hamburg, Germany; jianwei.zhang@uni-hamburg.de

**Keywords:** biped robot walking, knee-extended walking, inertia compensation, energy-efficient locomotion control

## Abstract

This paper investigates the locomotion of bipedal robots, with a focus on knee-extended walking. While knee joint extension is essential for efficient human walking, humanoid robots face challenges such as pose singularities, and traditional control methods often result in high joint velocities. To address these issues, static approaches have been proposed to achieve knee-extended walking. In this study, we present a pattern generation method based on the inertial linear inverted pendulum model (ILIPM) to simulate human arch motion. A quadrilateral foot structure and compliant control of the virtual leg are designed to enable knee-extended walking in biped robots. To enhance stability, we combine linear feedback control with an ankle joint strategy to correct the deviation of the divergent component of motion (DCM). Experimental comparisons were conducted across three scenarios: bent-knee walking, knee-extended walking without compliance control, and knee-extended walking with compliance control. The results show that knee-extended walking with compliance control results in the lowest energy consumption and minimizes the root mean square error (RMSE) of the center of mass (COM) velocity oscillations. Additionally, ILIPM-based walking experiments demonstrate smooth periodic oscillations of the COM trajectory with an amplitude of approximately 0.015 m. In the comparison of LIPM, Flywheel LIPM, and ILIPM, the ILIPM approach showed the least impact on the COM posture angle and angular momentum, leading to improved walking stability. Finally, DCM error correction experiments revealed that combining ankle joint control with linear feedback control provides the most effective correction of DCM errors.

## 1. Introduction

One of the primary objectives in humanoid robot research is to achieve seamless coexistence with humans. Consequently, humanoid robots are designed to emulate natural and flexible human movements, facilitating their smooth integration into society. However, traditional bipedal robots typically adopt a low pelvis height and bent-knee walking to ensure stability and avoid the knee-extended walking singularity. This approach increases torque demand on the knee joint, resulting in an ungraceful walking gait [1]. Mahdokht Ezat has concluded that knee-extended walking is the most efficient gait, especially when considering the angle associated with maximum torque at the knee joint [2].

To tackle this problem, Miura et al. [3], Ogura et al. [4], and Park [5] have all successfully achieved knee-extended walking of humanoid robots by combining a preset joint angle trajectory with adjustments to the center of mass (COM) height based on the zero moment point (ZMP) criterion. However, the variable COM height introduces an additional degree of freedom (DOF) at the waist, which can lead to knee joint singularities during knee-extended walking. Some researchers have proposed methods for knee-extended walking while maintaining a constant COM height. Kiss et al. [6] have employed linear springs to simulate muscle-tendon units and controlled the phase difference between hip and knee actuators to induce ankle joint movement for achieving knee-extended walking. Yoon et al. [7] have developed a foot device with a four DOFs parallel mechanism that adjusts the bipedal robot’s gait in the support phase to compensate for singularity issues caused by knee extension. However, controlling the vibration and rebound of such parallel mechanisms is challenging due to their stiffness-like behavior resembling pure springs. Huang et al. [8] have proposed a COM trajectory with horizontal position constraint to achieve desired knee extension posture, effectively resolving kinematic conflicts associated with knee extension gait by decoupling the centroid locus into the sagittal plane and transverse plane motion components. Nevertheless, the horizontal constraint limits the dynamic adaptability of the robot.

In this study, we investigate the implementation of knee-extended walking in bipedal robots through foot structure design. Initially, a quadrilateral structure foot was developed to regulate the height and pitch angle of the quadrilateral using two drive motors. This design mimics the biomechanical properties of the human foot arch to compensate for variations in COM height resulting from knee-extended walking.

Next, we explore the control of leg compliance to achieve a more energy-efficient and human-like knee-extended walking gait. Geyer et al. [9] demonstrated the importance of compliant legs in obtaining fundamental walking mechanics, such as ground reaction force (GRF) patterns, and introduced a spring–mass model for both walking and running. Jerry Pratt et al. [10] controlled the dynamic walking of bipedal robots by simulating virtual mechanical components, without relying on complex sensor systems. In this paper, we integrate the spring-damper model into the virtual leg control system of the robot to generate a Cartesian-based walking pattern without requiring pre-defined or redesigned knee joint trajectories.

Furthermore, in order to achieve efficient walking, we propose a walking pattern generation method based on the inertial linear inverted pendulum model (ILIPM). In this paper, we introduce the concept of COM moment of inertia derived from the invariant COM height to enhance the adaptability of the robot during knee-extended walking, thereby filling the gap between the linear inverted pendulum model (LIPM) and real robot dynamics. Additionally, we effectively correct the error of divergent component of motion (DCM) through a combination of linear feedback control and ankle joint strategy.

The subsequent sections of this paper are structured as follows: Section 2 presents the structural design of the quadrilateral foot and discusses both forward and inverse kinematics of the leg. In Section 3, we propose a method for simulating the human foot arch to regulate robot leg compliance, thereby achieving energy-efficient knee-extended walking. Section 4 introduces a pattern generator based on the ILIPM. Finally, our conclusions are presented in Section 5.

## 2. Novel Cable-Driven Mechanism for Bipedal Robots

### 2.1. Mechanism Description for Robot

The foot of a bipedal robot currently incorporates various designs, including single-point feet, single DOF joint module feet, and double DOFs joint module feet [11,12,13]. Its primary function is to absorb shock upon ground contact in order to maintain stable walking and standing balance. However, unlike the human foot arch, which possesses both rigidity and flexibility, the foot of a bipedal robot cannot achieve this [14]. A common solution is to incorporate soft materials such as rubber on the sole to mimic slip resistance and shock absorption [15]. However, excessive softness can result in insufficient stiffness making it challenging for robots to maintain stability. Conversely, increased hardness significantly reduces shock absorption capability. The flexed-knee design reduces reliance on the hip joint and creates a redundant angle between the thigh and calf, compared to the straight-leg design. This redundant angle helps absorb impacts during walking, providing shock cushioning and reducing joint stress. Therefore during walking, bipedal robots flex their legs by adjusting knee joint flexion angle to simulate spring-like shock-absorbing effects. Meanwhile, traditional knee-extended robots assume an unfavorable posture for transmitting force when extended, relying solely on structural components to withstand impact forces. Inspired by the arch structure of the human foot arch, we have devised a bipedal robot foot arch. By adjusting the bending of the foot arch, effective ground-buffering is achieved, resulting in a walking gait with increased knee extension that more closely resembles human walking patterns.

**Figure 1:**
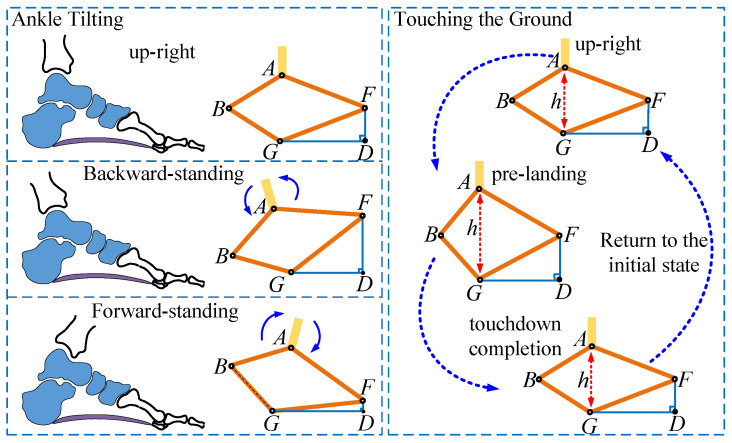
Foot arch structure design diagram.

The schematic of the bipedal robot foot arch in this paper, shown in Figure 1, innovatively demonstrates a quadrilateral mechanism design. The quadrilateral mechanism consists of two rocker arms, AF and AB, which are driven by motors to rotate around their respective axes. When both rocker arms rotate together, the system maintains their relative positions, allowing the ankle to swing forward and backward, mimicking the natural ankle motion in human gait. When both rocker arms rotate downward simultaneously, the angle between them decreases, causing the four-bar mechanism to bend. This results in a greater vertical bending of the foot, forming an arch-like shape. Conversely, when the rocker arms rotate upward, the angle between them increases, reducing the foot’s vertical bending and returning it to a straight position. By precisely controlling the rotation angles of the rocker arms, the degree of foot arch bending can be adjusted, producing a cushioning effect during ground contact. This cushioning helps reduce impact forces while walking, making the movement smoother and more natural. In this design, the motor is fixed to the thigh, and power is transmitted via a rope transmission system to the rotating disc at the ankle joint. The rotating disc then transfers the motor’s rotational motion to the rocker arms. Through this quadrilateral structure, the foot can achieve flexible bending and cushioning effects, improving the robot’s stability and adaptability in various environments.

### 2.2. Hardware Description

**Figure 2:**
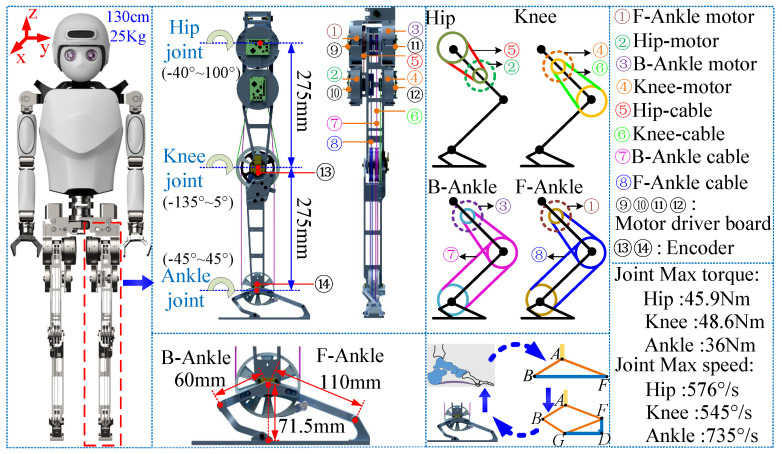
Robot leg structure diagram, which includes the range of motion of the hip, knee, and ankle joints, as well as the drive mode and maximum torque speed parameters of each motor in the joint. The serial number and component correspond by color.

The independently developed and innovatively designed biped robot platform is depicted in Figure 2. The bipedal robot has a height of 1.3 m and a weight of 25 kg. The robot possesses a total of 28 DOFs, with 10 DOFs allocated to its legs. In order to optimize the precision of the dynamic model while minimizing the leg moment of inertia, an innovative methodology was employed by symmetrically distributing 10 drive motors corresponding to the leg joints on both sides of the COM in the coronal plane. Additionally, rope drive technology was implemented to actuate the hip, knee, and ankle joints, effectively reducing the mass of each leg to 3.4 kg. Specifically, motors ① and ③ actuate the foot joints to form an arch structure, while motors ② and ④ independently control the hip and knee joints. Notably, the robot operates on the Linux operating system, with each joint equipped with an absolute encoder. Furthermore, we find the robot’s COM using the diagonal balance method, the inertial measurement unit (IMU) is mounted at the COM, and all motors are configured in position control mode to ensure optimal control outcomes [16].

### 2.3. Kinematics Analysis

**Figure 3:**
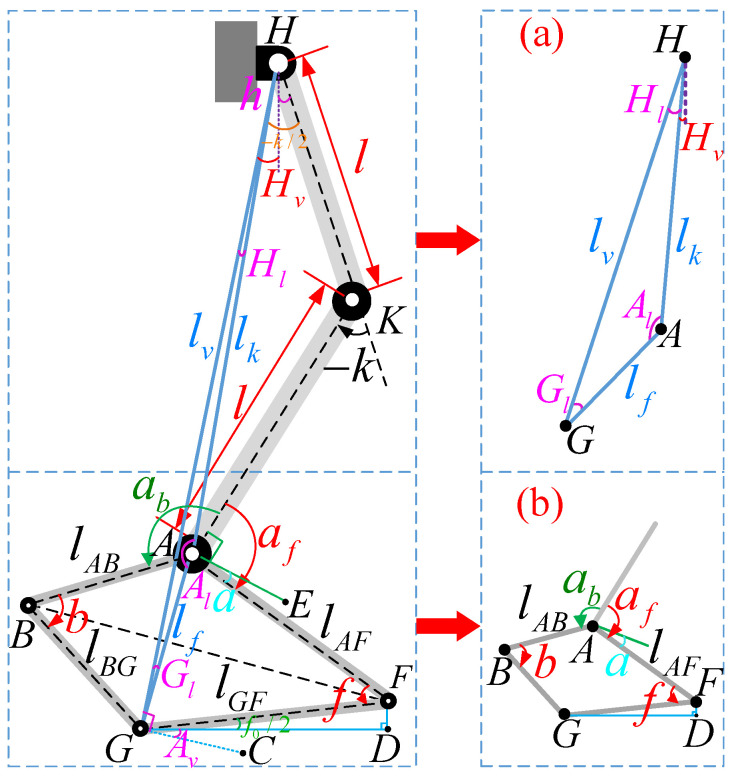
A schematic diagram shows leg motion analysis, using different letters and colors to represent angle and length. This visual representation illustrates the relationship between angle and length in leg motion analysis.

The leg structure diagram of the robot in this paper is shown in Figure 3, with *H* for the hip joint, *K* for the knee joint, and *A* for the ankle joint. The foot is represented by quadrilateral ABGF, where both sides $l_{AB}$ and $l_{BG}$ have a length of 0.06 m, and both sides $l_{AF}$ and $l_{GF}$ have a length of 0.11 m. The thigh and shank are denoted by HK and KA, respectively, with a fixed length of 0.275 m each. The virtual leg space is defined by the △ HAG, where its sides $l_{v}$, $l_{k}$, and $l_{f}$ correspond to the angles $H_{l}$, $A_{l}$, and $G_{l}$, respectively. The △ GF*D* is determined by structural design with ∠ FG*D* equal to $f_{0}/2$ (10°), which remains constant during movement. During the robot’s walking process, its foot is controlled to maintain stable ground contact by fixing the posture of △ GF*D*, ensuring reliable foot placement. HG is perpendicular to GC. ∠CGD equals $A_{v}$. KA is perpendicular to AE.

(a) Forward kinematics

In this paper, we employ forward kinematics (FKs) to plan the movement of the supporting leg and provide feedback on the contact point through joint angles. We utilize motor encoders to obtain precise feedback on hip joint angle *h*, knee joint angle −k, ankle joint angles *a* and *f* of the robot as input parameters to calculate the virtual leg space parameters.

Figure 3a illustrates the kinematic relationship of the virtual leg space. Here, $l_{k}$ represents the line connecting the hip joint and ankle joint ($l_{k}=2l\cos\left(-\frac{k}{2}\right)$), while $l_{f}$ denotes the arch height ($l_{f}=2l_{AF}\sin\left(\frac{f}{2}\right)$). Additionally, $A_{l}$ signifies the angle formed by $l_{k}$ and $l_{f}$ ($A_{l}=2\pi -\left(\frac{\pi }{2}-\frac{f}{2}\right)-\left(-\frac{k}{2}\right)-a_{f}$), whereas $l_{v}$ corresponds to the length of the virtual leg that requires control ($l_{\nu }=\sqrt{{l_{k}}^{2}+{l_{f}}^{2}-2l_{k}l_{f}\cos A_{\nu }}$).

The angle formed by $l_{k}$ and $l_{v}$ is denoted as $H_{l}$ ($H_{l}=asin\left(\frac{l_{f}}{l_{\nu }}\sin A_{l}\right)$), while the angle formed by $l_{v}$ and $l_{f}$ is represented as $G_{l}$ ($G_{l}=\pi -A_{l}-H_{l}$). The vertical rotation angle of $l_{k}$ is indicated as $H_{v}$ ($H_{v}=h-\left(-\frac{k}{2}\right)-H_{l}$). The control is based on the contact surface GD being considered as the horizontal plane, and the rotation angle of BF is denoted as $A_{v}$ ($A_{\nu }=G_{l}+\left(\frac{\pi }{2}-\frac{f}{2}\right)+\frac{f_{0}}{2}-\frac{\pi }{2}$).

Figure 3b represents the movement relationship of the quadrilateral feet. *a* represents the motion information of the ankle joint. $a_{f}$ represents the angle between the calf and the toe ($a_{f}=a+\frac{f}{2}+\frac{\pi }{2}$), $a_{b}$ represents the angle between the calf and the heel ($a_{b}=\frac{b}{2}-a+\frac{\pi }{2}$), *b* represents the angles of the quadrilateral foot ($b=2arcsin\left(\frac{l_{AF}}{l_{AB}}\sin\left(\frac{f}{2}\right)\right)$).

By performing FK calculations, we calculate virtual leg length $l_{v}$, corresponding angle $H_{v}$, and angle $A_{v}$. Subsequently, we map joint motion onto virtual leg movement to achieve precise control over robotic leg movements.

(b) Inverse kinematics

The inverse kinematic (IK) algorithm uses foot position and posture as inputs to calculate joint angles. In gait control, IK is employed to determine the target joint angles based on desired locomotion trajectories. This study applies IK to plan the swinging leg motion by incorporating virtual leg space ($l_{v}$, $H_{v}$, and $A_{v}$) to solve for the hip joint angle (*h*), knee joint angle (−k), and ankle joint angles (*a* and *f*). These virtual leg space values are derived from the robot’s initial pose and gait model. By analyzing the motion trajectory of the virtual leg during each movement cycle and using gait data, these values are then used in the inverse kinematics calculations to compute the joint angles, enabling precise control of the robot’s gait.

Firstly, we establish the functional relationship of the virtual leg space △HAG in Figure 3a, which is a known quantity. Determine *k* ($k=-2\arccos\left(\frac{l_{k}}{2l}\right)$) based on the side length $l_{k}$ of △HAG and derive *f* ($f=2\arcsin(\frac{l_{f}}{2l_{AF}})$) from $l_{f}$. Then, obtain values for both $a_{f}$ ($a_{f}=G_{l}-\frac{f}{2}$) and *a* ($a=a_{f}-\frac{f}{2}-\frac{\pi }{2}$) through *h* ($h=H_{v}+H_{l}+\left(-\frac{k}{2}\right)$) and *k*.

## 3. Compliance Control Strategy Based on the Biomechanics of Arch Structure

Humans can utilize the central neural system to continuously adapt the compliance characteristics of the ankle joint during walking or jumping, effectively absorbing the impact force through the arch and tendon units to exhibit a natural and seamless gait and improve walking efficiency [17]. However, when employing motor-driven legged robots, they often encounter significant impacts upon contact with the ground. These impacts result in substantial reverse currents being generated by the motors, which can lead to damage to the robot’s driving components. Without compliance control on the bipedal robot’s legs, its gait may become stiff and uncoordinated, compromising the smoothness of its movement. To address these issues, traditional bipedal robots with bent-knee walking have employed compliance control using a virtual spring damping system in their knee joints to absorb GRF [18]. However, for the same mechanical structure, bipedal robots with a flexed-knee walking design generally consume more energy compared to those with a knee-extended walking design [19,20]. In this paper, we propose implementing a virtual spring damping system on the virtual leg that leverages the height changes of a quadrilateral foot to buffer against GRF. This approach better aligns with human movement patterns by simulating the biomechanics of arch structures.

**Figure 4:**
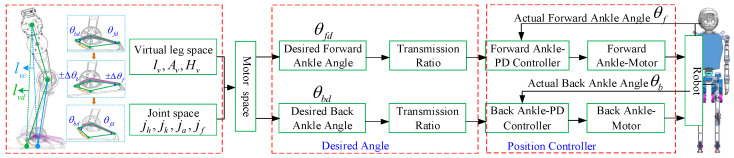
The compliant control strategy schematic diagram.

In the given scenario of a desired ankle joint angle, the robot encounters deviations in actual angles caused by external forces upon contact with the ground. These deviations affect the hip and knee joint angles, thereby influencing the overall leg position. To ensure that each joint adheres to the desired trajectory despite these interferences, we propose introducing a virtual leg ($l_{v}$) concept to compensate for this error. We regulate the length of $l_{v}$ to adjust GRF during robot–ground contact. By virtualizing both spring and damper elements on $l_{v}$, we enable energy storage and release through springs while facilitating energy absorption and dissipation through dampers, effectively controlling system vibrations, as depicted in Figure 4. We equate the virtual leg $l_{v}$ to a spring and maintain a stable gait by adjusting the spring’s elastic coefficient $k_{p}$ and damping coefficient $k_{d}$.

According to the law of energy conservation, when the robot comes into contact with the ground, there is a change in torque of $l_{v}$, which is equal to the torque generated by the quadrilateral structure on the foot. This torque in the quadrilateral structure is transmitted through two driving motors at the ankle joint. The relationship can be established as shown in (1). The subscripts *f* and *b* denote the toe and heel, respectively. $\Delta \theta_{f}$ and $\Delta \theta_{b}$ represent variations in both actual angles and target angles of two ankle joints—one located at the front and another at the back of the foot.

(1) $$\left[\begin{array}{c} F_{lx}\\ F_{ly} \end{array}\right]\cdot \Delta l_{v}=\left[\begin{array}{c} \tau_{f}\\ \tau_{b} \end{array}\right]\left[\begin{array}{cc} \Delta \theta_{f} & \Delta \theta_{b} \end{array}\right]$$

The relationship between virtual leg length variation ($\Delta l_{v}$) and joint angle variation ($\Delta \theta_{f}$, $\Delta \theta_{b}$) is described by (2).

(2) $$\Delta l_{v}=\left[\begin{array}{cc} \frac{\partial l_{v}}{\partial \theta_{f}} & \frac{\partial l_{v}}{\partial \theta_{b}} \end{array}\right]\left[\begin{array}{c} \Delta \theta_{f}\\ \Delta \theta_{b} \end{array}\right]=J\left[\begin{array}{c} \Delta \theta_{f}\\ \Delta \theta_{b} \end{array}\right]$$

The compliance controller is designed on the virtual leg according to (3), where $l_{v}$ corresponds to the current values $\theta_{f}$ and $\theta_{b}$, and the subscript *d* represents the target values. Both $l_{v}$ and $F_{l}$ have components in the *x* and *y* directions. $k_{p1}$ and $k_{d1}$, respectively, represent the control gains.

(3) $$F_{l}=k_{p1}\left(l_{vd}-l_{v}\right)-k_{d1}{\dot{l}}_{v}$$

We used MATLAB (version 2023b; Natick, MA, USA) to simulate the joint rate variations of the robot’s legs, controlling the gait to maintain the foot sole parallel to the ground during walking. The relationship between the $F_{l}$ on the virtual leg and the torque ($\tau_{f}$ and $\tau_{b}$) applied at the ankle joint is described in (4), with additional details in (1) and (2).

(4) $$J^{T}\cdot F_{l}=\left[\begin{array}{c} \Delta \tau_{f}\\ \Delta \tau_{b} \end{array}\right]$$

The following statement is provided for deduction.

(5) $$k_{p}=\left[\begin{array}{c} k_{pf}\\ k_{pb} \end{array}\right],k_{d}=\left[\begin{array}{c} k_{df}\\ k_{db} \end{array}\right]$$

(6) $$\Delta \theta =\left[\begin{array}{c} \Delta \theta_{f}\\ \Delta \theta_{b} \end{array}\right],\dot{\theta }=\left[\begin{array}{c} {\dot{\theta }}_{f}\\ {\dot{\theta }}_{b} \end{array}\right]$$

The deduction of (7) can be obtained by combining (3) and (4), resulting in (8) and (9).

(7) $$J^{T}\cdot k_{p1}\cdot J\cdot \Delta \theta -J^{T}\cdot k_{d1}\cdot J\cdot \dot{\theta }=k_{p}\cdot \Delta \theta -k_{d}\cdot \dot{\theta }.$$

(8) $$k_{p}=J^{T}\cdot k_{p1}\cdot J$$

(9) $$k_{d}=J^{T}\cdot k_{d1}\cdot J$$

In this paper, we utilize the parameters $k_{p}$ and $k_{d}$ to fine-tune the force exerted on the virtual leg, thereby emulating the human foot arch. The objective of this adjustment is to rectify the error between the actual ankle joint angle and the target angle caused by external forces upon robot–ground contact.

## 4. Pattern Generation Based on ILIPM

### 4.1. Steady-State Control Model of COM

Currently, the prevailing trend in bipedal robot design is to minimize the weight of the legs and concentrate the driving system near the COM. However, this approach may lead to an increase in the moment of inertia around the ground reference frame. Specifically, knee-extended walking results in a stride length, but during the swing phase and knee extension, the robot’s mass distribution changes, which can significantly increase the moment of inertia around the ground axis [21]. This may affect the robot’s stability and control precision, particularly when rapid gait adjustments are needed during movement. Traditional biped robots often employ LIPM as a simplified model, which does not account for the moment of inertia [22]. In order to achieve efficient walking similar to humans, we propose a method for generating walking patterns based on ILIPM.

**Figure 5:**
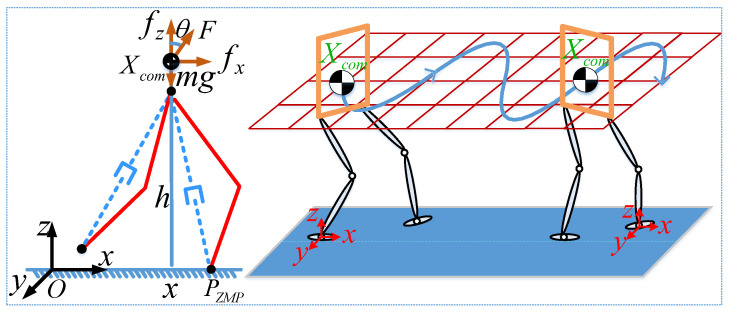
The simplified model of the robot. In the figure on the left, the red solid-line representation of the robot leg and the blue dashed-line depiction of the virtual leg. $X_{com}$ represents the position of the COM, θ denotes the angle of virtual leg, $F_{c}$ signifies the force acting on the COM, $p_{\mathrm{ZMP}}$ indicates the contact position, and *h* corresponds to the COM height.

The ILIPM for the robot is a simplified mathematical model employed to describe the dynamic behavior of the robot during walking, as illustrated in Figure 5.

By utilizing Newton’s second law and incorporating concepts such as the moment of inertia, equations describing the motion in a linear inverted pendulum (LIP) system can be derived based on principles of rotational dynamics, as demonstrated in (10) [23]. Within this framework, *I* represents the moment of inertia, it is related to the mass of the robot and the distribution of the mass with respect to the COM; $\ddot{\theta }$ represents angular acceleration at COM; *m* corresponds to COM mass; $l_{v}$ defines virtual leg length; and finally, *g* denotes gravitational acceleration. Furthermore, τ indicates the control torque exerted on COM.

(10) $$I\ddot{\theta }-mgl_{v}\sin\theta =\tau$$

The relationship between the length of the virtual leg ($l_{v}$) and the position of the COM (*x*) is described by (11).

(11) $$x=l_{v}\sin\theta \approx l_{v}\theta$$

(12) $$\ddot{x}=\left(mgx+\tau \right)\cdot \frac{l_{v}}{I}$$

The COM in the human body is typically located around the waist area. During walking, particularly brisk walking or running, rotational movement of the torso commonly occurs. Excessive or inappropriate moments of inertia can result in increased instability [24]. To withstand the frequent and intense shocks resulting from dynamic motion, biped robot design requires lightweight leg links and repositioning hardware such as motors closer to the COM in order to more closely resemble the LIPM. However, this increase in weight at the COM affects its moment of inertia and, consequently, impacts its motion trajectory. The LIPM, with a variable COM height, can effectively address the stability issues encountered during bipedal robot bent-knee walking [25]. However, it may lead to a singularity in the knee joint during knee-extended walking. The LIP Plus Flywheel Model introduces the moment of inertia of the COM by applying torque and frictional resistance to the flywheel [26]. However, the control of the hip joint is often transformed into an optimization problem in terms of providing flywheel torque and attitude control, thus adding complexity to the control system.

In this paper, compensating for the extra moment of inertia $I_{c}$ of the COM to solve the problem of moment of inertia caused by external force to ensure the stable walking of the robot. Then, the robot’s total moment of inertia becomes $I_{sum}=ml^{2}+I_{c}$. We can define $R_{I}=\sqrt{\frac{1}{mh^{2}+I_{c}}}$ and $\omega^{*}=R_{I}\sqrt{mgh}$ to introduce $I_{c}$ to balance out the external force disturbance derived from Newton’s second law ILIPM control equation for (13).

(13) $$\ddot{x}={\omega^{*}}^{2}\left(x-p_{\mathrm{ZMP}}\right)$$

### 4.2. Error Correction for DCM

The DCM is a control approach based on the relationship between the robot’s COM and the contact point [27]. It can be represented as a virtual point that describes the trajectory of the robot’s COM in space [28]. However, practical applications of DCM may introduce bias due to external interference or inherent limitations of the model itself, thereby compromising balance control. Current control strategies, such as the ankle joint strategy, have been shown to effectively adjust the ZMP, thereby influencing its balance [29]. However, although this strategy can effectively minimize the final value error of DCM, it does not provide a guarantee for maintaining a consistently small range of DCM errors throughout the motion. An alternative approach involves employing linear feedback control, which adjusts the DCM error based on a precise dynamic model and possesses the ability to promptly respond to variations in the DCM error [30]. However, the minimization of terminal error cannot be guaranteed by linear feedback control alone. Therefore, this study proposes a novel approach that synergistically combines linear feedback control with the ankle strategy to effectively achieve rapid and precise DCM error correction, leveraging their complementary advantages.

According to [31], DCM can be defined as (14).

(14) $$\xi =x+\frac{1}{\omega }\dot{x}$$

Based on (13), the dynamics of DCM can be derived as (15).

(15) $$\dot{\xi }=\omega^{*}\left(\xi -p_{\mathrm{ZMP}}\right)$$

The analytical solution for DCM is derived as (16), where $\xi_{T}$ is the DCM at the end of step time T, which is expressed as the DCM end-of-step. $\xi_{t}$ and $p_{t,\mathrm{ZMP}}$ are the current DCM and current ZMP.

(16) $$\xi_{T}=\left(\xi_{t}-p_{t,\mathrm{ZMP}}\right)e^{\omega^{*}\left(T-t\right)}+p_{t,\mathrm{ZMP}}\;\left(0\leq t\leq T\right)$$

Based on the ZMP criterion, the ankle torque is regulated to balance the CoM torque τ (in this paper, tau consists of the moment $\tau_{1}$ generated by the terminal error and the moment $\tau_{2}$ generated by the process error), effectively controlling the ZMP and ensuring the robot’s stability. To integrate the ankle strategy and linear feedback control strategy within a cohesive control framework, we employ ankle torque to manipulate the ZMP position and regulate the end-of-step error of DCM. As depicted in (17), $u_{0}$ represents the center of the stance foot, which remains constant throughout the current step phase.

(17) $$p_{\mathrm{ZMP}}=u_{0}+\frac{\tau }{mg}$$

The current error Δξ and end-of-step error $\xi_{T,\mathrm{err}}$ of DCM are represented by (18) and (19), respectively.

(18) $$\Delta \xi =\xi_{t}-\xi$$

(19) $$\xi_{T,\mathrm{err}}=\left(\Delta \xi -\frac{\tau_{1}}{mg}\right)e^{\omega^{*}\left(T-t\right)}+\frac{\tau_{1}}{mg}$$

The linear feedback control strategy focuses on utilizing the current error of the DCM to dynamically adjust the control output, aiming to effectively rectify future trajectory errors. The current error Δξ of DCM can be adjusted by control gain $k_{p}$. The fundamental control law is depicted in (20).

(20) $$\Delta \dot{\xi }=\omega^{*}\left(\Delta \xi +\frac{\tau_{2}}{mg}\right)=\left(1-k_{p}\right)\omega^{*}\Delta \xi$$

By adjusting the torque τ of the ankle joint, the robot’s DCM error can be effectively controlled.

(21) $$\tau =\tau_{1}+\tau_{2}$$

## 5. Results

**Figure 6:**
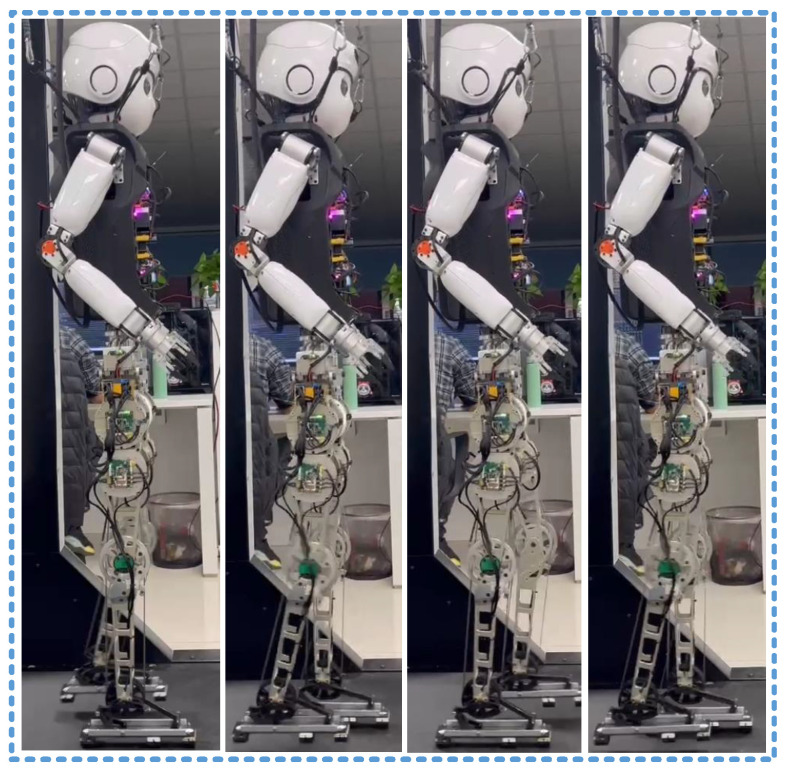
Real-world walking experiment.

We rigorously validated the effectiveness of our arch-type bipedal robot in knee-extended walking through a series of experiments conducted with the physical robot in real-world environments, as shown in Figure 6.

### 5.1. Virtual Leg Compliance Control

**Figure 7:**
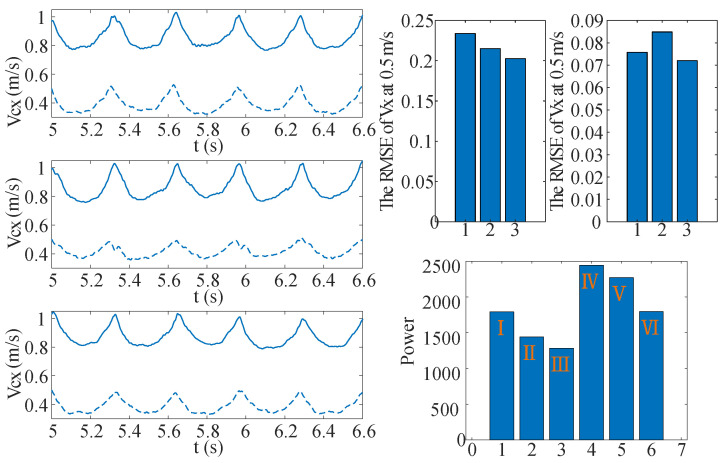
The three rows of graphs on the left represent the variation in the COM velocity (Vcx) for different walking gaits: case ➀ bent-knee walking, case ➁ knee-extended walking without compliance control, and case ➂ knee-extended walking with compliance control ($k_{pf}/k_{pb}=600$, $k_{pf}/k_{db}=50$), under set speeds of 0.5 m/s and 1 m/s. Dashed lines correspond to 0.5 m/s, and solid lines represent 1 m/s. Top right bar chart shows the RMSE of velocity for cases ➀, ➁, and ➂ at walking speeds of 0.5 m/s and 1.0 m/s. This is used to analyze the oscillation of the center of mass velocity and assess walking stability. The experimental results indicate that case ➂ performs the best at both walking speeds. Bottom right bar chart illustrates the power consumption for these gaits at two speeds. The first three columns correspond to 0.5 m/s for cases ➀, ➁, and ➂, respectively; the last three columns represent the same gaits at 1 m/s. This figure demonstrates that the knee-extended walking gait with compliance control is the most energy-efficient.

Humans efficiently walk through the natural compliance of leg joints and muscles, which involves multiple joints (such as ankles, knees, hips) and associated muscle groups rather than relying on a single joint. However, traditional knee joint-based bipedal robot compliance control fails to effectively store and release energy, resulting in higher energy consumption. To tackle this issue, we propose a compliance control method utilizing virtual legs that emulate the function of tendons as spring-like elements during human locomotion. The proposed approach facilitates the attainment of human-like compliance in robot legs, thereby significantly enhancing energy efficiency during locomotion. In this experiment, we will compare the performance of knee compliance control and virtual leg compliance control in terms of power output. Key measurements include torque and angular velocity at the hip, knee, and ankle joints. Torque is measured using current, and power output $P=\sum_{i}\tau_{i}\cdot \omega_{i}$ is calculated accordingly. The experimental results are shown in Figure 7.

In the left graph of Figure 7, disparities in COM velocity fluctuations among the three gaits at identical speeds are clearly visible. Specifically, bent-knee walking exhibits larger fluctuations in COM velocity, whereas knee-extended walking with compliance control demonstrates significantly reduced fluctuations. These fluctuations, characterized by sharp peaks and variations in the velocity curve, indicate short-term instability in the system, influenced by factors such as the robot’s response or environmental changes. While we have not quantitatively analyzed each fluctuation, we ensure the consistency and reliability of the data through rigorous experimental design and repeated measurements. The right bar chart shows the power requirements for various gaits at two different speeds. It is evident that knee-extended walking with compliance control consumes the least power at both speeds, highlighting its superior energy efficiency. Moreover, this gait’s ability to maintain stable performance in the face of external disturbances while minimizing energy loss due to environmental variations further underscores its robustness and adaptability.

### 5.2. Walking Experiment of ILIPM

**Figure 8:**
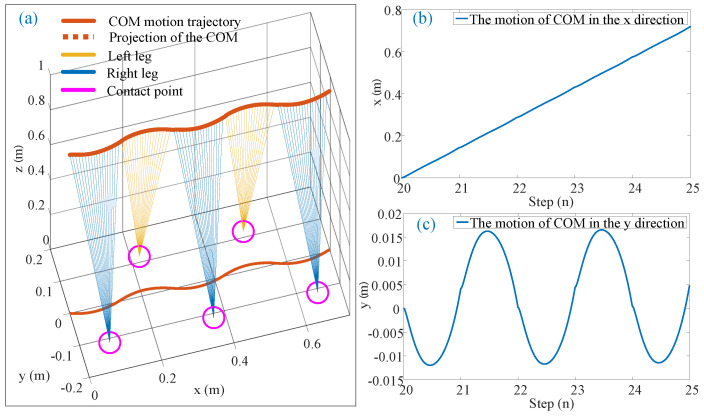
(**a**) The three-dimensional relationship between the COM and contact points; (**b**) the trajectory of the COM in the *x* direction from steps 20 to 25; (**c**) the trajectory in the *y* direction during the same time period. The experimental findings demonstrate a smooth periodic oscillation between both feet for the COM trajectory with an amplitude approximately equaling 0.015 m.

To validate the impact of compensating for the centroid’s rotational inertia during the robot’s walking process using the ILIPM on gait stability and performance, experiments were conducted on a robot platform, as shown in Figure 8. During the experiment, the robot’s forward speed command is set at 0.5 m/s, each step length is 0.144 m, lateral step width is 0.112 m.

### 5.3. Comparison of LIPM, Flywheel LIPM, and ILIPM

**Figure 9:**
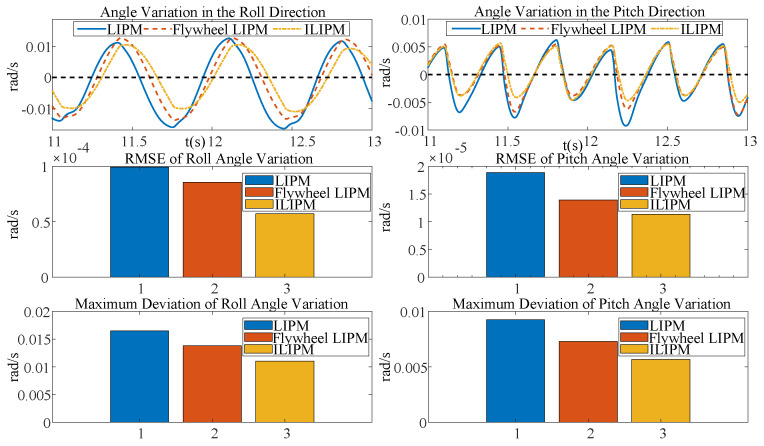
The figure illustrates the angular variations of the COM in roll and pitch directions under three control models, (LIPM, Flywheel LIPM, and ILIPM). The above figures depict the temporal changes in angles. The middle figures display the RMSE, which represents the overall deviation. The lower figures present the maximum deviations. It is observed that LIPM exhibits a higher RMSE sum deviation and poor stability, whereas ILIPM demonstrates a lower RMSE sum deviation and good stability.

**Figure 10:**
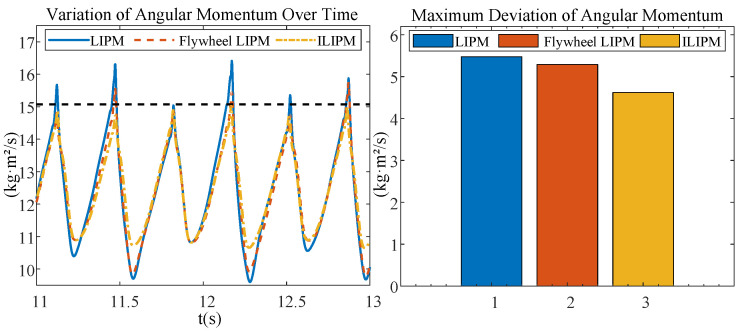
The figure depicts the variation in angular momentum of the robot, with a reference value of 15.075, derived from $L_{y}=v_{x}*m*h$. The results indicate that LIPM exhibits a higher maximum deviation, implying instability in controlling angular momentum. Conversely, ILIPM demonstrates a lower maximum deviation, suggesting enhanced stability and reliability in regulating angular momentum during forward walking.

In this experiment, we compare the performance of three models: the LIPM, the Flywheel LIPM, and the ILIPM, under a constant forward velocity set at $v_{x}$ = 0.9 m/s. To ensure a fair comparison of the three gait models, we maintained identical hardware configurations across all experiments, varying only the control strategies. The robot’s mechanical structure and actuation system remained unchanged, while the LIPM, Flywheel LIPM, and ILIPM gaits were implemented through distinct control methodologies. Specifically, the Flywheel LIPM incorporated a flywheel to enhance angular momentum, whereas the ILIPM utilized inertial compensation to improve gait stability. This approach guarantees that any observed differences in gait performance stem solely from the control strategies. The experimental findings are depicted in Figure 9 and Figure 10.

The ILIPM surpasses the other two models in minimizing angular variations in the roll and pitch directions, as well as fluctuations in angular momentum, as illustrated in Figure 9 and Figure 10. ILIPM effectively reduces gait fluctuations through precise regulation of the COM’s attitude angle and angular momentum. This control strategy enhances postural stability by actively responding to external disturbances via real-time estimation and compensation mechanisms.

### 5.4. Ankle Joint Control: Assessing Disturbance Resistance

The experiment compares three control strategies aimed at reducing DCM error in a biped robot under external interference. A thrust of 10 Ns was applied, and the trajectories of COM, DCM, and contact points were recorded. Figure 10 demonstrates that the combination of linear feedback with ankle joint control strategy effectively rectifies DCM errors and enhances balance.

**Figure 11:**
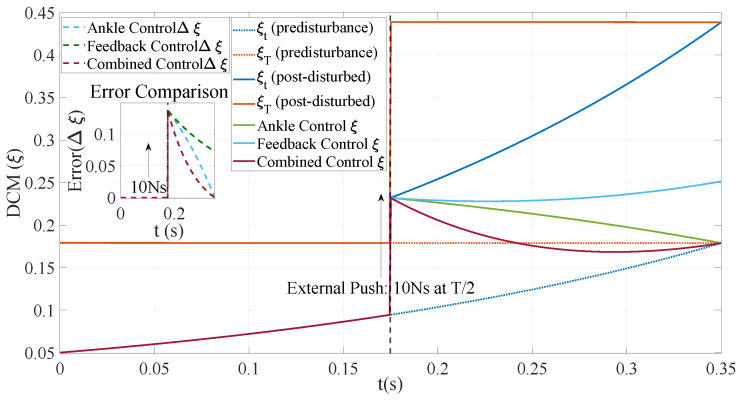
DCM control strategies and error analysis. The ankle control (green solid line) effectively tracks the end position of the DCM but exhibits a significant error during tracking. The linear feedback control (light blue solid line) demonstrates less error during tracking but has a poor tracking effect at the end position. The combined control (red solid line) integrates the strengths of both approaches to minimize error during tracking and at the end position. The comparison of errors in the subgraph further illustrates that the initial error of ankle joint control is larger while the terminal error is smaller. Conversely, the initial error of linear feedback control is small, with a large terminal error. In contrast, combination control yields minimal error and achieves rapid convergence throughout the entire process.

The ankle joint control demonstrates superior stability at the end position, as illustrated in Figure 11, while the linear feedback control exhibits a faster response to disturbances. These two strategies are synergistically integrated to optimize and rectify errors at different stages throughout the entire control process. This combination effectively mitigates cumulative error and ensures simultaneous enhancement of system response speed and terminal stability. Consequently, it enables effective correction of DCM errors and significantly enhances system balance and stability.

## 6. Discussion

This paper represents a significant step forward in the field of bipedal robotics, specifically addressing the challenges and solutions associated with knee-extended walking—a critical aspect of humanoid locomotion that closely emulates human walking dynamics. The core contribution of our study lies in the development and validation of a novel pattern generation method based on the ILIPM and effectively correcting the error of DCM through a combination of linear feedback control and ankle joint strategy. Through the innovative design of a quadrilateral foot structure and implementation of compliant control for the virtual leg, we have successfully emulated the human foot arch, significantly enhancing stability and efficiency in bipedal locomotion. This approach effectively enables knee-extended walking in biped robots, overcoming longstanding challenges associated with pose singularities and high joint velocities that have hindered progress in this field.

Yu Jianjun et al. proposed a teaching method that leverages human walking data, utilizing imitation learning to enable humanoid robots to replicate human walking patterns, thereby achieving bipedal robot gait [32]. Beomyeong Park et al. employed model predictive control (MPC) to generate the COM trajectory in real-time, enabling natural gait patterns with heel-to-toe and toe-to-heel footfalls [5]. However, while both methods enhance gait naturalness, they also increase the complexity of the algorithms and control systems. This paper introduces an innovative compensation mechanism based on the ILIPM, using a quadrupedal foot structure to adjust the robot’s COM moment of inertia and achieve humanoid gait in bipedal robots. In Chapter 5, we conducted a systematic comparison of three models: LIPM, Flywheel LIPM, and ILIPM. The experimental results demonstrated that the ILIPM exhibited the highest stability in the robot’s walking gait. Compared to existing methods, the proposed ILIPM algorithm not only effectively achieves humanoid gait but also significantly simplifies control, making it well-suited for conventional bipedal robots with mechanical structures. This innovation offers a new approach to bipedal robot gait control, providing considerable value in enhancing both gait stability and control simplicity. In contrast to previous studies, such as Masoumeh Safartoobi et al. [33], who conducted detailed dynamic modeling and numerical simulations to explore passive walking bipedal robot models with compliant legs and analyzed the effects of various parameters on gait stability (without physical experiments), we applied a compliance control strategy using virtual legs. This approach effectively improved the cushioning effect during ground contact in physical experiments. The results showed that robots with leg compliance control significantly reduced power consumption and exhibited less oscillation in the COM velocity, leading to a more stable gait compared to robots without compliance control.

In future research, we plan to further optimize the robot’s structure by incorporating foot force sensors, which will facilitate the collection of real force data. This will enable more precise gait optimization and a comprehensive comparison of mechanical forces, ultimately enhancing the model’s performance and accuracy. Additionally, future work will focus on developing a more efficient and compact control algorithm for the proposed mechanical structure. The goal is to fully harness the humanoid characteristics of bipedal robots, driving significant improvements in both energy efficiency and stability.

## 7. Conclusions

In this paper, we introduce an innovative approach to achieving knee-extended walking in bipedal robots using the ILIPM. By designing a quadrilateral foot structure and implementing compliant control of the virtual leg, we successfully emulate the human foot arch, effectively addressing challenges such as pose singularities commonly encountered in traditional control methods. Additionally, by integrating linear feedback control with an ankle joint strategy, we effectively correct the DCM error, thereby enhancing the robot’s stability. Experimental results show that knee-extended walking with compliance control leads to lower energy consumption and reduced COM velocity oscillations. Moreover, our ILIPM-based walking experiments demonstrate smooth COM trajectory oscillations, with ILIPM offering superior stability compared to models such as LIPM and Flywheel LIPM. Future research will focus on applying this methodology to diverse terrains, further optimizing energy efficiency, and conducting long-term stability tests to ensure the robustness and reliability of the approach. 

## Data Availability

The data presented in this study are available on request from the corresponding author.
